# Preparation of Hierarchically Structured Perlite NPs/Metformin–Co (II) System to Catalyze the Green Synthesis of Pyrazolopyranopyrimidines

**DOI:** 10.1002/open.202500407

**Published:** 2025-10-06

**Authors:** Jalal Reihani Shurbakhlu, Leila Moradi, Abdulhamid Dehghani

**Affiliations:** ^1^ Department of Organic Chemistry Faculty of Chemistry University of Kashan Kashan P.O. Box 87317‐51167 Iran

**Keywords:** green chemistry, heterogeneous catalyst, multicomponent reaction, perlite nPs/Met‐co(II), pyrazolopyranopyrimidine

## Abstract

This study presents an innovative heterogeneous nanocatalytic system designed through a multistep synthetic approach involving the surface functionalization of perlite nanoparticles (Perlite NPs/Met‐Co(II)). Comprehensive characterization using fourier transform infrared spectroscopy (FTIR), X‐ray diffraction, field‐emission scanning electron microscopy, energy‐dispersive X‐ray analysis (EDX), elemental mapping, thermogravimetric analysis, and brunauer‐emmett‐teller (BET) analyses confirms the successful formation of a hierarchically structured catalyst. The catalyst enables efficient one‐pot multicomponent synthesis of pyrazolopyranopyrimidines in water (100 °C, 5 mg loading), yielding 87–95% in 30–60 min. The presented method adhering to green principles and also recyclability (6 cycles) and synergistic performance provides an atom‐economic platform for sustainable heterocycle synthesis.

## Graphical abstract

This study presents an innovative heterogeneous nanocatalytic system based on perlite nanoparticles (Perlite NPs/Met‐Co(II)). Comprehensive characterization using FTIR, XRD, field‐emission scanning electron microscopy, EDX, elemental mapping, TGA, and BET analyses confirms the hierarchically structure of nanocatalyst. The catalyst enables green and efficient one‐pot multicomponent synthesis of pyrazolopyranopyrimidines in water adhering to green principles.

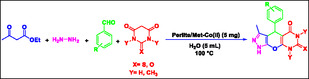

## 1 Introduction

Perlite, a type of rhyolites, is a natural glass with a distinctive pearl‐like structure. Its physical properties, including refractive index and specific weight, are similar to those of obsidian. Unique characteristics of perlite include its very low density, high porosity, heat resistance, chemical neutrality, and resistance to insects and fungi.^[^ ^1^ ^–^ ^3^ ^]^ Due to its special properties, perlite can be used in various industries, including construction, agriculture, casting, filtration, paint, and coatings.^[^ ^4^, ^5^ ^–^ ^6^ ^]^ Nowadays, perlite is considered a superior support for the stabilization of biocatalysts. Perlite's porous structure with high specific surface area makes it a promising catalyst carrier. This natural mineral compound is used in many catalytic processes due to its unique physical and chemical properties and cost‐effectiveness. Perlite‐based catalysts are produced by attaching the desired catalyst to the pearlite surfaces. This attachment can be accomplished using methods like impregnation, precipitation, and chemical or physical processes.^[^ ^2^ ^,^ ^7^, ^8^, ^9^ ^–^ ^10^ ^]^ Heterogeneous perlite‐based catalysts can be used in the pharmaceutical, petrochemical, and air and water purification industries.^[^ ^11^, ^12^ ^–^ ^13^ ^]^ Given the great advantages of perlite, it can be utilized for the development of new catalysts and the improvement of catalytic processes especially in multicomponent reactions (MCRs).^[^ ^14^, ^15^ ^–^ ^16^ ^]^

In recent years, MCRs have emerged as a powerful and versatile tool for efficient and flexible synthesis in modern organic chemistry.^[^ ^17^ ^,^ ^18^ ^]^ These reactions offer several advantages, including the creation of new and diverse compounds in a minimum number of steps, high atom economy, simple reaction pathways, short reaction times, high selectivity, and excellent yields. Given these benefits, MCRs are considered as a green and environmentally friendly approach for accessing a library of complex molecules, heterocyclic systems, and biologically active compounds.^[^ ^19^, ^20^ ^–^ ^21^ ^]^ Recent advances in green MCRs have focused on water‐based protocols and natural supports for heterocyclic synthesis, aligning with principles of atom economy and waste minimization. ^[^ ^22^ ^,^ ^23^ ^]^ For instance, catalyst‐free or bioderived systems have enabled efficient construction of nitrogen heterocycles under mild conditions.^[^ ^24^ ^]^

Heterocyclic compounds are fundamental building blocks in countless synthetic and natural substances. These structures have broad applications in supramolecular chemistry, pharmacology, electronics, agriculture, and optics.^[^ ^25^ ^,^ ^26^ ^]^ Moreover, they are crucial intermediates in the synthesis of various natural and pharmaceutical products. As a result, the development and synthesis of heterocyclic compounds have become a major focus for researchers.^[^ ^27^ ^,^ ^28^ ^]^

**Figure 1:**
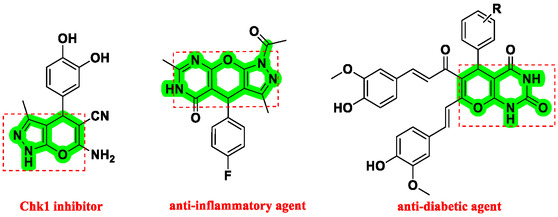
Representatives of pyranopyrazole, pyranopyrimidine, and pyrazolopyranopyrimidine compounds with pharmacological activity.

Nitrogen‐containing heterocyclic compounds which are a significant portion of natural and synthetic substances have proven their valuable role in modern organic synthesis and medicinal chemistry. These compounds possess a wide range of pharmacological and biological activities, such as anticancer, antifungal, antibacterial, anti‐inflammatory, and antimalarial properties (**Figure** **1**).^[^ ^29^, ^30^ ^–^ ^31^ ^]^ Pyrazolopyranopyrimidines are fused nitrogen‐containing heterocyclic compounds characterized by a planar, aromatic scaffold with polar functional groups, including carbonyl (C=O) and amine (NH) moieties. These structures impart solubility in polar solvents such as water and ethanol, facilitating their synthesis under green conditions. Their physicochemical properties, such as moderate melting points (200–250 °C) and stability in aqueous media, enhance their suitability for catalytic processes. Recent studies confirm their diverse pharmacological activities, including anticancer and anti‐inflammatory effects, supported by molecular interactions with biological targets.^[^ ^32^, ^33^, ^34^, ^35^, ^36^ ^–^ ^36^ ^]^ Some of them also show antimalarial, antifungal, pain‐relieving, and weed‐killing properties.^[^ ^37^, ^38^ ^–^ ^39^ ^]^ To synthesize these compounds, researchers used a range of catalysts, including ZnFe_2_O_4_/GA,^[^ ^32^ ^]^ meglumine,^[^ ^40^ ^]^ SDS,^[^ ^41^ ^]^ ChCl:Urea,^[^ ^42^ ^]^ Nano‐ZnO,^[^ ^43^ ^]^ oleic acid,^[^ ^44^ ^]^ halloysite clay nanotubes,^[^ ^45^ ^]^ Fe_3_O_4_@cellulose,^[^ ^46^ ^]^ magnetized Water,^[^ ^47^ ^]^ graphene oxide,^[^ ^48^ ^]^ oxidized multiwalled carbon nanotubes (OMWCNTs),^[^ ^49^ ^]^ DABCO,^[^ ^50^ ^]^ MNS‐ionic liquid,^[^ ^51^ ^]^ SBA‐PR‐SO_3_H,^[^ ^52^ ^]^ TiO_2_ NWs,^[^ ^53^ ^]^ and silica nanoparticles. .^[^ ^54^ ^]^ Despite these advances, many reported catalysts suffer from drawbacks such as high loading requirements, organic solvent dependency, or limited recyclability, leading to increased waste and costs.^[^ ^32^ ^,^ ^40^ ^–^ ^54^ ^]^ Recent green MCRs emphasize natural catalyst supports and water media, but few integrate low‐cost minerals like perlite with metal‐organic complexes for enhanced synergy.^[^ ^55^, ^56^ ^–^ ^57^ ^]^ This highlights the need for innovative, recyclable systems that combine atom economy with superior performance. Unlike previous catalysts limited by synthetic supports or harsh conditions, this work's novelty stems from the hierarchical structure enabling faster reactions (30–30 min) and higher recyclability (6 cycles) in water, promoting broader adoption in ecofriendly organic synthesis.

**Scheme 1:**
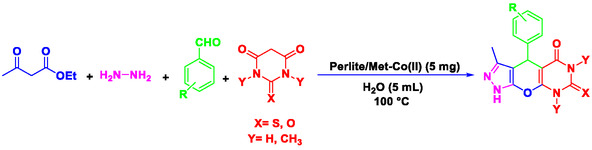
Preparation of pyrazolopyranopyrimidine derivatives using Perlite NPs/Met‐Co(II).

The utilization of water as solvent in the synthesis of heterocyclic compounds offers a sustainable and efficient approach to minimize the waste generation and reduces the reaction time and energy consumption.^[^ ^45^ ^,^ ^58^ ^]^ Nowadays, chemists are concentrating on the preparation of heterogeneous catalytic systems that are efficient, cost‐effective, and easy to recover, particularly for the synthesis of heterocyclic and pharmaceutical compounds. In this study, pyrazolopyranopyrimidine derivatives were synthesized using a novel, efficient, ecofriendly, and reusable nanocatalyst (Perlite NPs/Met‐Co(II)) (**Scheme** **1**). The reaction was carried out in water under thermal conditions, leading to high yield of pyrazolopyranopyrimidine products in short times. This method offers a more efficient and environmentally friendly approach compared to traditional methods. The novelty lies in the first‐time use of perlite nanoparticles functionalized with metformin‐Co(II), offering enhanced porosity, low‐cost sourcing, and exceptional recyclability beyond existing catalysts.

## 2 Results and Discussion

### 2.1 Characterization of Perlite NPs Perlite NPs/Met‐Co(II)

**Scheme 2:**
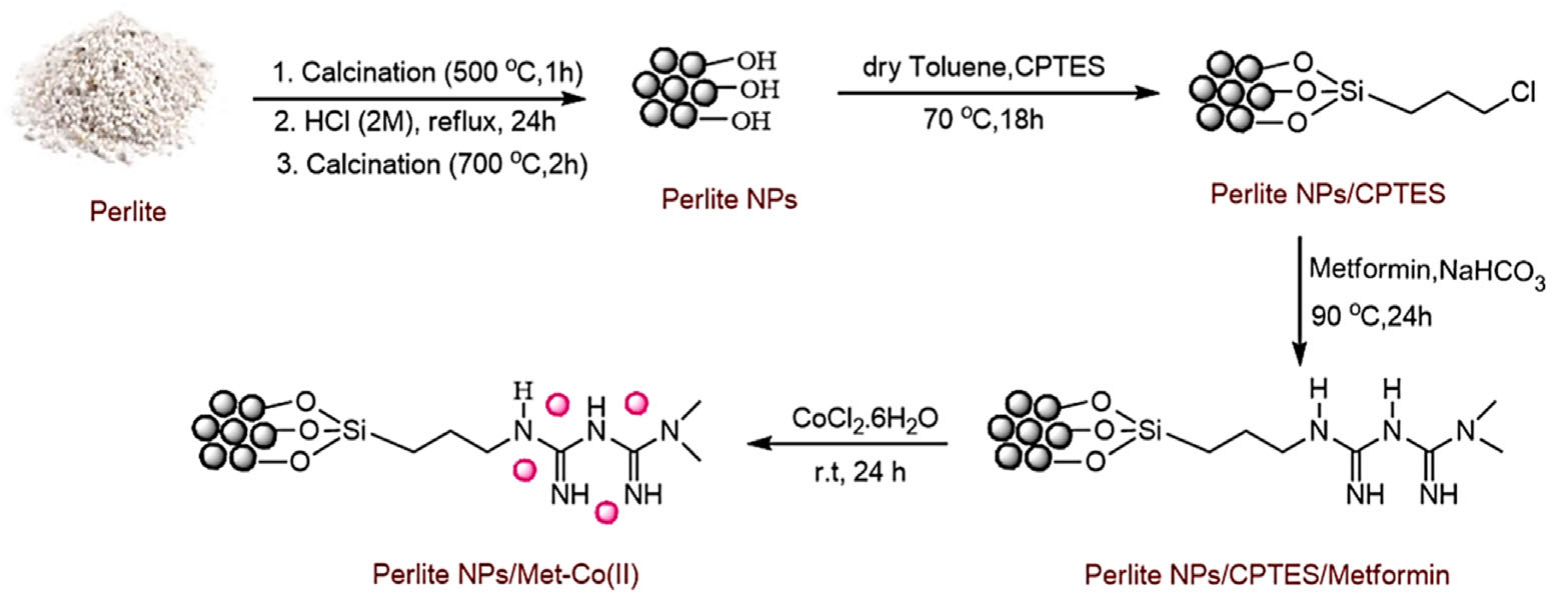
The synthesis steps of the Perlite/CPTES/Met‐Co(II)

The Perlite NPs/Met‐Co(II) nanocatalyst was synthesized as shown in **Scheme** **2** and characterized using FTIR, X‐ray diffraction (XRD), FESEM, EDX, elemental mapping, TGA, and BET techniques. Analytical results demonstrated effective functionalization of the Perlite NPs with CPTES and Metformin /Co (II) species.

### 2.2 XRD Analysis

**Figure 2:**
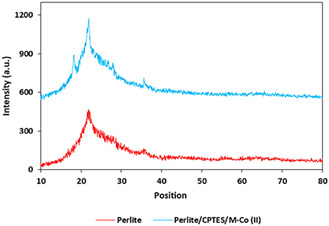
XRD pattern of Perlite NPs and Perlite NPs/Met‐Co(II).

XRD analysis was employed to investigate the crystalline structure of both pristine Perlite NPs and the Perlite NPs/Met‐Co(II) catalyst (**Figure** **2**). The diffraction pattern of Perlite NPs (Figure 2a) confirms their amorphous structure.^[^ ^7^ ^,^ ^8^ ^]^ Upon functionalization (Figure 2b), the composite maintains the amorphous structure while revealing subtle crystalline features attributable to the incorporated cobalt species. XRD pattern of the catalyst shows low‐intensity peaks at 2*θ* of 16°, 32°, 35°, and 42°, consistent with reference patterns for cobalt chloride (JCPDS card no. 72–2408),^[^ ^59^ ^]^ though significantly attenuated due to the following: 1) highly dispersed cobalt ions within the perlite matrix (≤5 wt % loading), 2) strong metal–support interactions distorting the Co coordination environment, and 3) peak overlap with residual crystalline domains of perlite. The observed peak broadening and intensity reduction suggest successful incorporation of cobalt ions into interstitial sites of the amorphous perlite framework, which creates active sites in catalyst.^[^ ^60^ ^,^ ^61^ ^]^

### 2.3 FTIR Analysis

**Figure 3:**
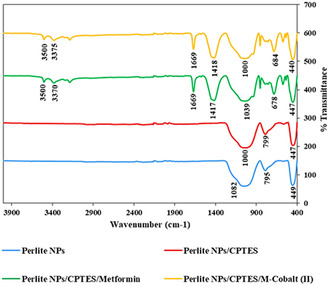
FTIR spectra of Perlite NPs, Perlite NPs/CPTES, Perlite NPs/CPTES/Met, and Perlite NPs/Met‐Cobalt (II).

FTIR spectroscopy was employed to confirm the functionalization of the Perlite NPs. The FTIR spectra of Perlite NPs (a), Perlite NPs/CPTES (b), Perlite NPs/CPTES/Met (c), and Perlite NPs/Met‐Co(II) (d) are shown in **Figure** **3**. In the FTIR spectrum of Perlite NPs (Figure 3a), absorption bands at 1082 cm^−1^ and 795 cm^−1^ correspond to Si–O–Si and Si–O–M stretching vibrations, while the band at 449 cm^−1^ is associated with Si–O bending vibrations.^[^ ^60^ ^]^ For Perlite NPs/CPTES (Figure 3b), the absorption band at 799 cm^−1^ is from the stretching vibration of C‐Cl after the addition of CPTES.^[^ ^61^ ^]^ In FTIR spectrum of Perlite NPs/CPTES/Met (Figure 3c), the stretching vibrations at 3370 cm^−1^ and 3500 cm^−1^ are from the stretching vibrations of NH groups, and also the absorption band at 1417 cm^−1^ is related to the bending vibrations of NH_2_. The stretching vibration at 1669 cm^−1^corresponds to C= N, and the absorption band at 1039 cm^−1^ is related to C–N vibrations. The absorption band at 678 cm^−1^ is attributed to N−H out‐of‐plane bending.^[^ ^62^ ^]^ Finally, in the FTIR spectrum of Perlite NPs/Met‐Co(II) (Figure 3d), it can be observed that the FTIR spectrum of the Perlite NPs/CPTES/Met‐Co(II) remains similar to Perlit NPs/CPTES/Met, with the absorption band in the 1418 cm^−1^ region corresponding to the NH_2_ and cobalt stretching vibrations showing overlapping features. Therefore, to further confirm the stabilization of cobalt (II) on the surfaces of the nanocatalyst, additional analyses such as EDS and elemental mapping were done.

### 2.4 FESEM Analysis

**Figure 4:**
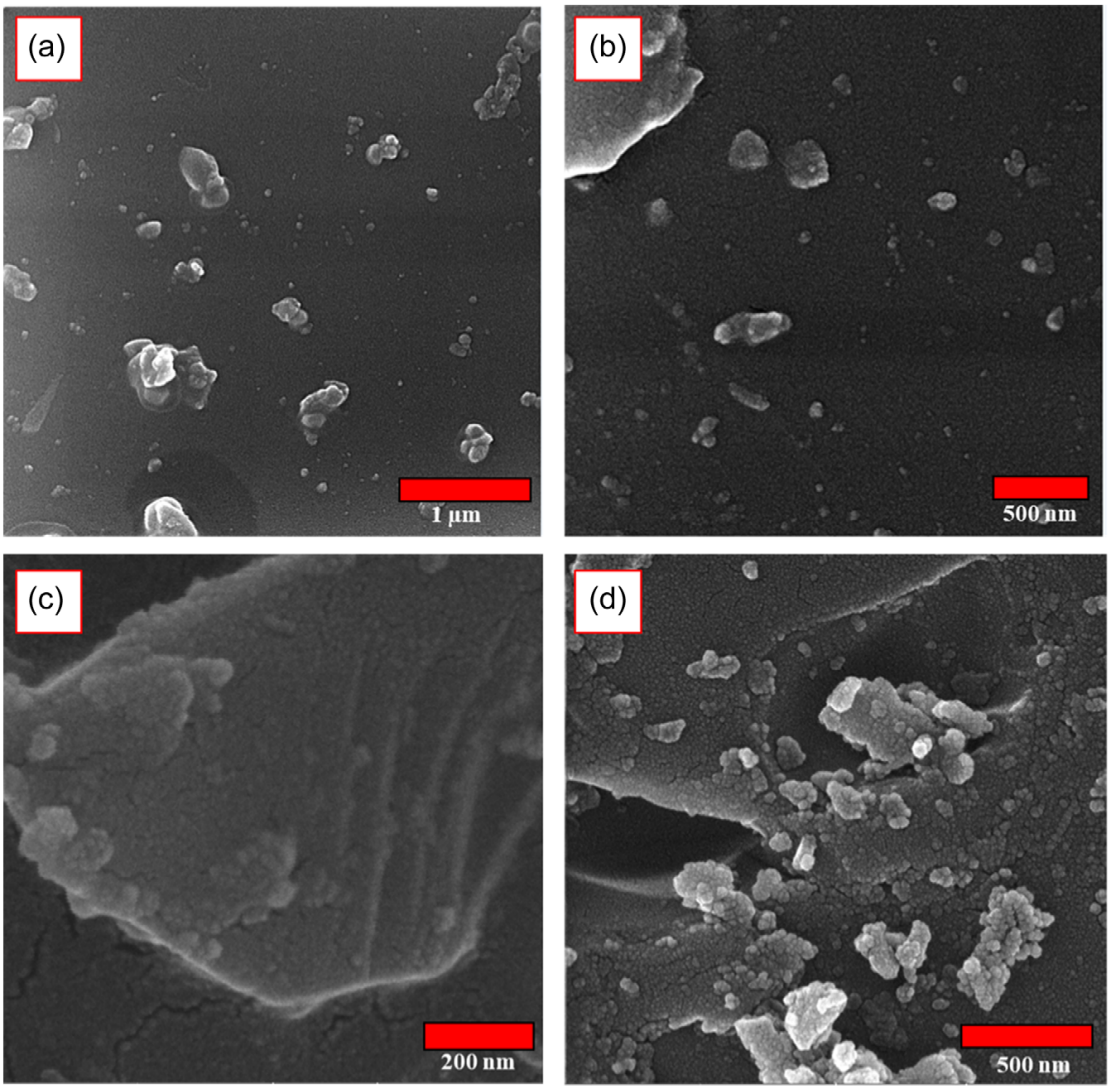
a–b) FESEM images of Perlite NPs and c–d) Perlite NPs/Met‐Co(II).

**Figure** **4** presents a comparative FESEM analysis of Perlite NPs and the prepared catalyst, providing key morphological insights. The images of the pristine Perlite NPs (Figure 4a,b) confirm their successfully synthesis at the nanoscale.^[^ ^7^ ^,^ ^8^ ^]^ A comparison between the raw and the functionalized Perlite NPs reveals well‐dispersed particles with preserved interparticle porosity and surface topology, indicating that the modification process did not change the spherical morphology of nanostructures. The observed surface roughening in the composite can be attributed to the successful grafting of CPTES/Met‐CO(II) on the surfaces of Perlite NPs.

### 2.5 EDX & Elemental Mapping Analysis

**Figure 5:**
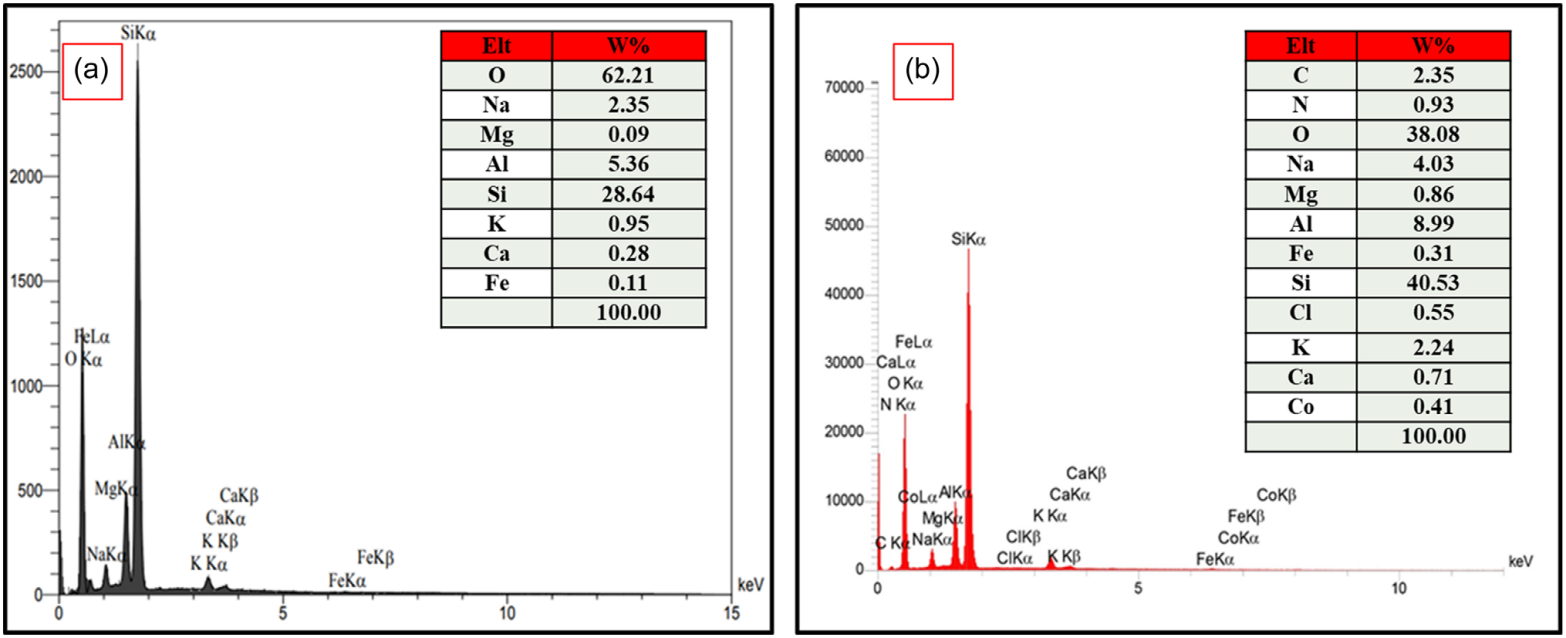
EDX analysis of a) Perlite NPs and b) Perlite NPs/Met‐Co(II).

EDX analysis was performed to characterize the elemental composition of the nanostructures (**Figure** **5**a,b). The spectrum in Figure 5a reveals characteristic peaks of Si, Al, K, Ca, Fe, Na, Mg, and O elements in the Perlite NPs, and Figure 5b demonstrates successful functionalization due to the presence of C, Cl, Co, and N elements in the Perlite NPs/Met‐Co(II) composite. The detection of additional elements confirms the surface modification of Perlite NPs while preserving the original mineral structure.

**Figure 6:**
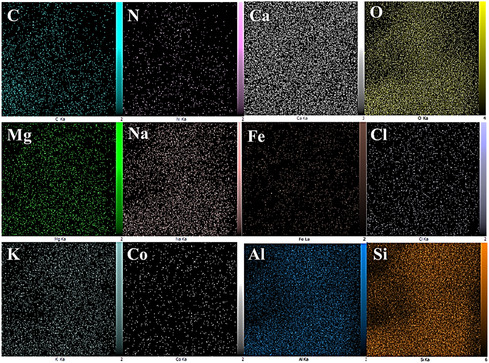
Elemental mapping images of Perlite NPs/Met‐Co(II).

**Figure** **6** shows the elemental mapping analysis of Perlite NPs/Met‐Co(II), revealing uniform distribution of all components throughout the structure. The elemental mapping results confirm successful functionalization, with well‐dispersed cobalt active sites on Perlite NPs structure. This homogeneous elemental distribution demonstrates effective synthesis of an integrated hybrid material, ensuring optimal catalytic site accessibility and maintaining structural integrity, crucial for enhanced catalytic performance.

**Figure 7:**
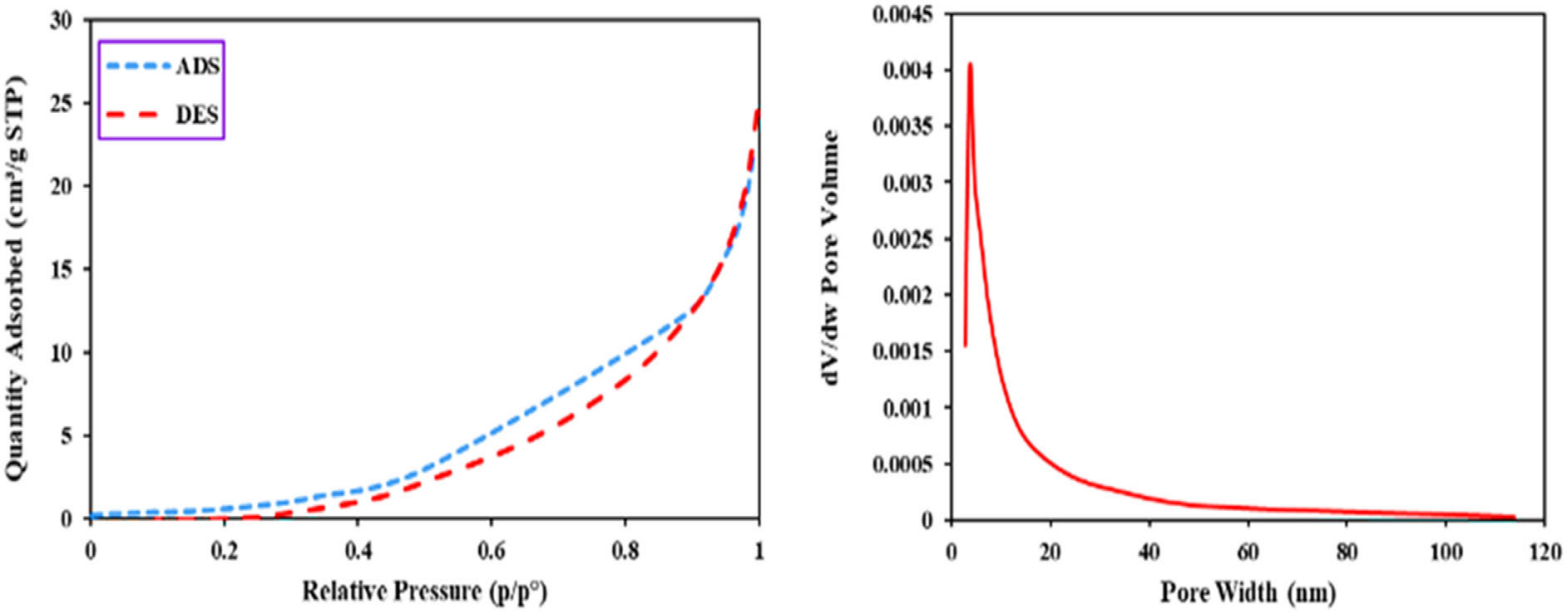
The N_2_ adsorption–desorption (ADE & DES) and pore size distribution of freshly synthesized Perlite NPs/Met‐Co(II).

### 2.6 BET Analysis Perlite

Nitrogen physisorption measurements revealed the porous structure of the synthesized Perlite NPs/Met‐Co(II) nanocatalyst (**Figure** **7**). The specific surface area of the prepared catalyst was obtained as 4.9199 m^2 ^g^−1^. The textural properties were further elucidated by Barrett–Joyner–Halenda (BJH) pore size distribution analysis, which identified a mesoporous structure with an average pore width of 9.6443 nm. The adsorption–desorption isotherm profile displayed characteristic type IV behavior according to IUPAC classification, with a pronounced H3 hysteresis loop observed at a relative pressure (*P*/*P*_0_) range of 0.45–0.95. This hysteresis morphology suggests the presence of slit‐shaped mesopores arising from platelet‐like particle aggregation. The absence of saturation plateau at high *P/P*_0_ indicates multilayer adsorption on macropore surfaces, consistent with the material's hierarchical porosity. Notably, the relatively low surface area correlates with the dense cobalt coordination complex grafting onto the perlite framework, while the significant pore diameter facilitates reactant diffusion during catalytic applications.

### 2.7 TGA Analysis

**Figure 8:**
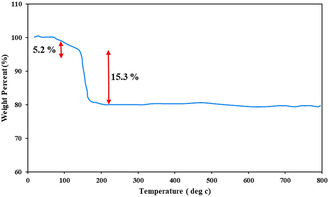
TGA diagram of Perlite NPs/Met‐Co(II).

Thermogravimetric analysis (TGA) was conducted to evaluate the thermal behavior and structural stability of the synthesized Perlite NPs/Met‐Co(II) nanocatalyst. The thermal decomposition profile, as shown in **Figure** **8**, exhibited two characteristic weight loss stages. The first mass loss (5.2%) between 50 and 150 °C is attributed to the removal of physically adsorbed water or solvent molecules from both surface sites and internal pore networks. As the temperature increased, a sharp 15.3% weight loss was observed from 150 to 230 °C. This significant mass loss corresponds to the thermal decomposition of the CPTES linker and metformin attached on Perlite NPs surfaces, demonstrating the successful modification of Perlite NPs.

### 2.8 Investigation of Perlite NPs/Met‐Co(II) Efficiency as Catalyst for the Synthesis of Pyrazolopyranopyrimidines

A systematic investigation was conducted to establish optimal reaction conditions for the synthesis of pyrazolopyranopyrimidines using 4‐nitrobenzaldehyde, ethyl acetoacetate, hydrazine hydrate, and barbituric acid as model reaction. The study examined three critical parameters: solvent, catalyst loading, and reaction temperature (results are detailed in **Table** **1**). Solvent screening revealed water as the superior reaction medium, achieving maximum product yield (entry 4), while aprotic polar solvents (DMSO, DMF) and pure alcohols (EtOH, MeOH) showed inferior performance. Notably, solvent‐free conditions produced negligible conversion (entry 7), highlighting the essential role of aqueous medium in facilitating the reaction. Catalyst evaluation was done with an uncatalyzed reaction which shows only trace products after extended duration (entry 8). The optimal catalyst loading was determined to be 0.005 g and higher (0.007 g) and provided no significant improvement (entry 10). Temperature studies show 100 °C can be considered as optimal temperature. Lower temperatures (60, 80 °C) show lower yield of product. The identified ideal conditions (0.005 g catalyst in water at 100 °C) afforded excellent yield within 35 min, representing a significant improvement over the conventional method. This optimization demonstrates the catalyst's ability to mediate efficient transformations in environmentally benign aqueous media as well as minimizing the energy and reaction time.

**Table 1:** Optimization of the reaction conditions for the synthesis of pyrazolopyranopyrimidines.

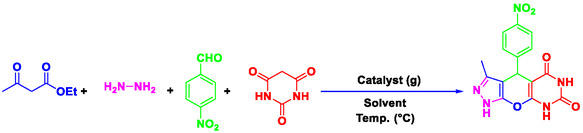					
Entrya)	Solvent	Catalyst [mg]	Time [min]	Temp. [°C]	Yieldb) [%]
1	EtOH	5	35	100	73
2	H_2_O/EtOH (1:1)	5	35	100	80
3	MeOH	5	35	100	45
4	H_2_O	5	35	100	95
5	DMSO	5	35	100	38
6	DMF	5	35	100	23
7	–	5	35	100	35
8	H_2_O	–	35	100	Trace
9	H_2_O	3	35	100	83
10	H_2_O	7	35	100	95
11	H_2_O	5	35	r.t.	Trace
12	H_2_O	5	35	60	45
13	H_2_O	5	35	80	65

a) Reaction conditions: ethyl acetoacetate (1 mmol), hydrazin hydrate (1 mmol), 4‐nitrobenzaldehyde (1 mmol), barbituric acid (1 mmol), solvent (5 mL);

b) Isolated yield.

To comprehensively evaluate the synthetic potential of presented MCRs, several aromatic aldehydes (1.0 mmol) (with electron‐withdrawing and electron donating groups), ethyl acetoacetate (1.0 mmol), hydrazine hydrate (1 mmol), and barbituric acid derivatives (1 mmol), were reacted under the previously established optimal conditions (**Table** **2**). Notably, the reaction shows the superior conversion rates with aldehydes bearing electron‐withdrawing substituents compared to their electron‐donating analogs.

**Table 2:** Synthesis of pyrazolopyranopyrimidine derivatives in the presence of Perlite NPs/Met‐Co(II)a).

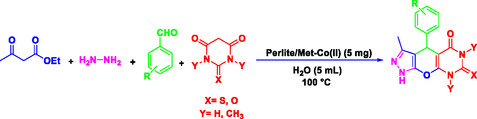		
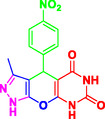 5a, 30 min, 95% TONb) = 2730, TOFc) = 5460 h^−1^	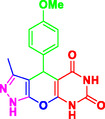 5b, 50 min, 92% TON = 2644, TON = 3148 h^−1^	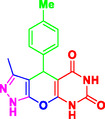 5c, 50 min, 92% TON = 2644, TOF = 3148 h^−1^
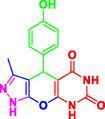 5d, 30 min, 89% TON = 2558, TOF = 5116 h^−1^	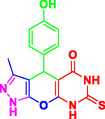 5e, 50 min, 89% TON = 2558, TOF = 3045 h^−1^	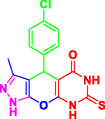 5f, 40 min, 88% TON = 2529, TOF = 3775 h^−1^
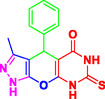 5g, 35 min, 91% TON = 2615, TOF = 4509 h^−1^	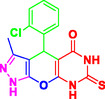 5h, 60 min, 85% TON = 2443, TOF = 2443 h^−1^	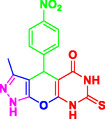 5i, 30 min, 93% TON = 2672, TOF = 5344 h^−1^
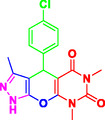 5j, 30 min, 87% TON = 2500, TOF = 5000 h^−1^	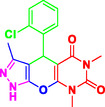 5k, 45 min, 87% TON = 2500, TOF = 3333 h^−1^	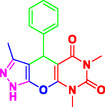 5l, 30 min, 92% TON = 2644, TOF = 2588 h^−1^

a) Reaction conditions: barbituric acid (or 1,3‐dimethylbarbituric acid, or thiobarbituric acid) (1 mmol), ethyl acetoacetate (1 mmol), aromatic aldehydes (1 mmol), hydrazin hydrate (1 mmol), Perlite NPs/Met‐Co(II) (5 mg), H_2_O (5 mL), 100  °C;

b) Turnover Number (TON) is calculated as moles of product formed per mole of catalytically active Co(II) sites, determined by EDX analysis (0.41 wt% Co).

c) Turnover Frequency (TOF) = TON / time (h).

### 2.9 Proposed Mechanism for the Synthesis of Pyrazolopyranopyrimidines

**Scheme 3:**
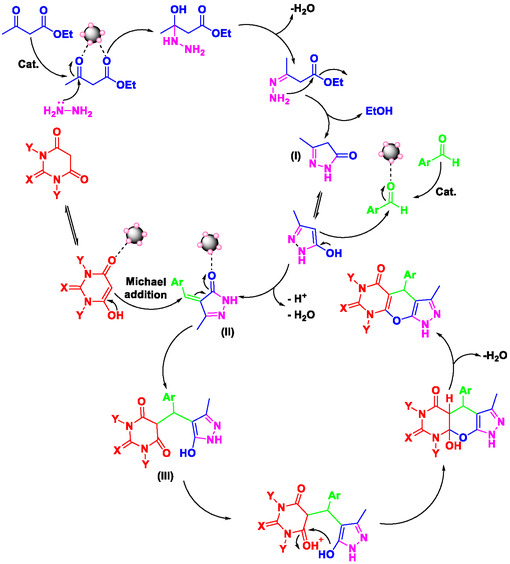
Suggested mechanism for the four‐component synthesis of pyrazolopyranopyrimidine derivatives in the presence of Perlite NPs/Met‐Co(II).

The plausible mechanism for the synthesis of pyrazolopyranopyrimidines in the presence of Perlite NPs/Met‐Co(II) is depicted in **Scheme** **3**. First, the catalyst‐activated condensation of hydrazine hydrate and ethyl acetoacetate was done to form 3‐methyl‐1H‐pyrazol‐5(4H)‐one as intermediate **I**, after elimination of ethanol and water (through two‐step condensation reaction). Second, Perlite NPs/Met‐Co(II) activated the carbonyl group of aldehyde through the coordination of Co(II) with oxygen atom of carbonyl to facilitate the nucleophilic attack of the intermediate **I** to yield the Knoevenagel product as intermediate **II.** The obtained intermediate then undergoes Michael addition reaction with barbituric acid, to produce intermediate **III**. Finally, the resulting enol undergoes intramolecular cyclization, to form the pyrazolopyranopyrimidine product after removal of H_2_O.

### 2.10 Investigation of the Catalyst Reusability

**Figure 9:**
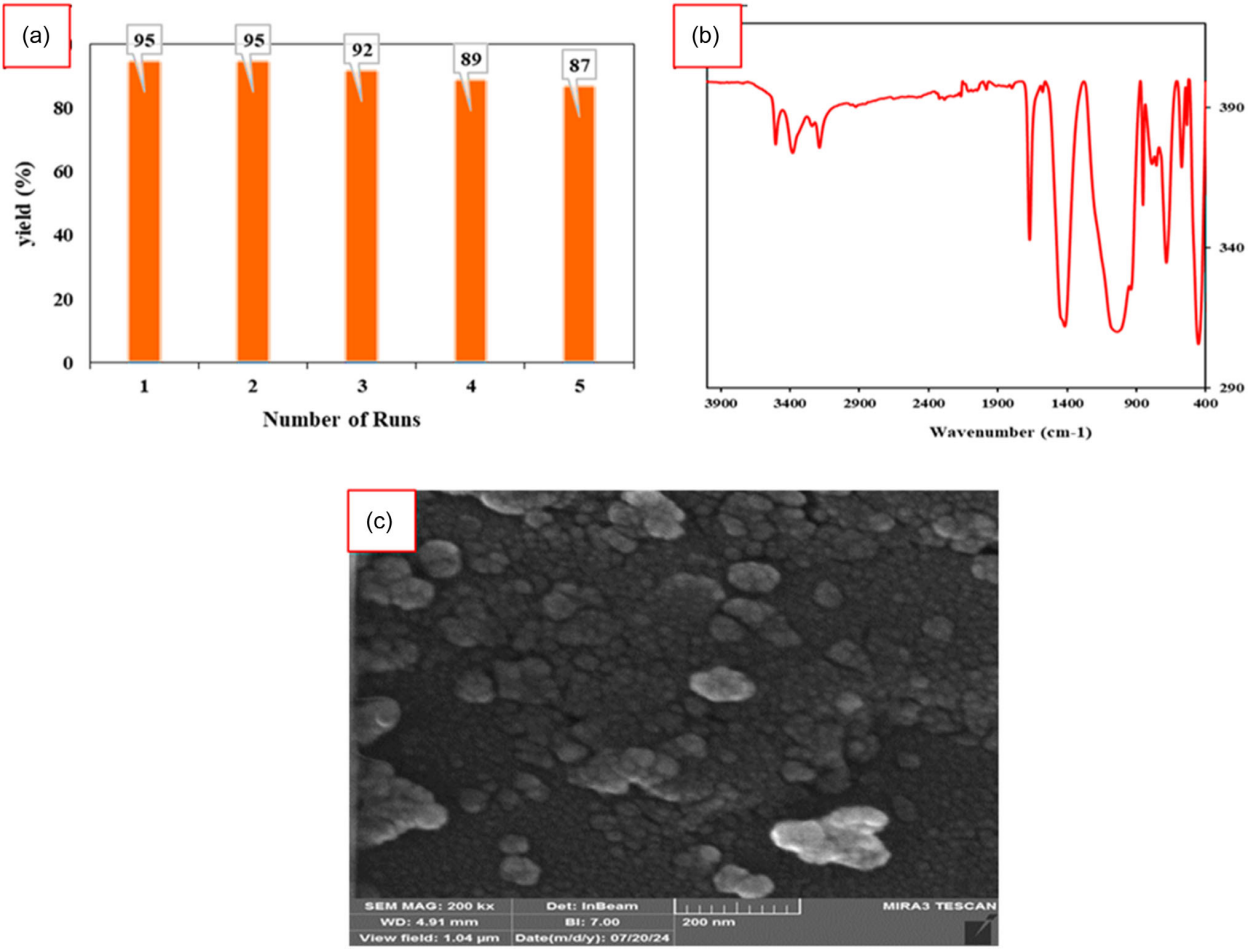
a) Study on the recoverability of the nanocatalyst, b) FTIR, and c) FESEM image of recovered catalyst after fifth run.

In line with green chemistry principles, the reusability and facile separation of the Perlite NPs/Met‐Co(II) nanocatalyst were evaluated through consecutive reaction cycles involving ethyl acetoacetate, hydrazine hydrate, benzaldehyde, and barbituric acid under identical conditions. As illustrated in **Figure** **9**a, the catalyst maintained high activity over five successive runs, demonstrating a low decrease in efficiency, which confirms its stability and suitability for sustainable applications. Characterization via FTIR and FESEM techniques confirmed the structural and morphological integrity of the reused nanocatalyst after repeated runs (Figure 9b,c). FTIR analysis verified the preservation of all functional groups, to confirm any significant degradation. Also, FESEM imaging revealed no morphological changes. Additionally, the ease of catalyst preparation and separation highlight its practical advantages, making Perlite NPs/Met‐Co(II) a promising, ecofriendly, and recyclable heterogeneous catalyst for sustainable organic transformations.

### 2.11 Comparison of the Perlite NPs/Met‐Co(II) Efficiency with Other Reported Catalyst

To emphasize the advantages and innovative nature of our catalytic approach, **Table** **3** provides a comparison with reported methods for the synthesis of pyrazolopyranopyrimidine derivatives (**5a**). While the documented catalysts possess certain benefits, in most cases, the employment of Perlite NPs/Met‐Co(II) as catalyst offers reduced reaction times and increased the product yields, making it as a more suitable option for the synthesis of pyrazolopyranopyrimidines compared to previously reported methods. In comparison of the Perlite NPs/Met‐Co(II) system with our prior ZnFe_2_O_4_/GA catalyst,^[^ ^32^ ^]^ (entry 11) 98% yield was achieved after 15 min at 100 °C with 0.01 g ZnFe_2_O_4_/GA. The presented method (Perlite NPs/Met‐Co(II) uses half the catalyst loading (0.005 g), reducing resource use by 50%, while maintaining yields of 87–95% over 30–60 min. The hierarchical perlite structure enhances 6 cycles recyclability (vs. 4 cycles in^[^ ^32^ ^]^), due to stable catalyst active sites. Compared to meglumine (20 mol%, 120 min) and Nano‐ZnO (0.015 g, 45 min), our approach offers lower loading, shorter or comparable times, and superior recyclability, reinforcing its green chemistry advantages and scalability. While the documented catalysts possess certain benefits, the employment of Perlite NPs/Met‐Co(II) as catalyst offers reduced reaction times and increased the product yields, making it a more suitable option for the synthesis of pyrazolopyranopyrimidines compared to previously reported methods.

**Table 3:** Comparative study of Perlite NPs/Met‐co(II) with reported methods for the synthesis of pyrazolopyranopyrimidines.

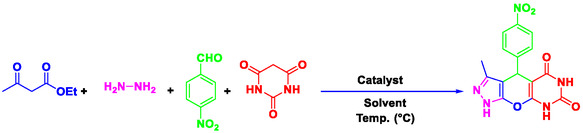				
Entrya)	Reaction condition	Time [min]	Yield [%]	Reference
1	Oleic acid, H_2_O, 100 °C	20	87	[44]
2	Fe_3_O_4_@cellulose nanocomposite (5 mg), H_2_O, r.t	40	90	[46]
3	OMWCNTs, EtOH/H_2_O, Reflux	65	90	[49]
4	MNPs@DABCO^+^Cl^−^ (0.01 g), solvent‐free, 80 °C	7	94	[51]
5	TiO_2_ NWs (10 mol%), EtOH/H_2_O (1:1)	55	91	[53]
6	Cu^2+^@MSNs‐(CO_2_^–^)_2_ (1.3 mol%), H_2_O, r.t	75	85	[54]
7	TEDA/IMIZ‐BAIL@UiO‐66 (0.05 g), EtOH, Reflux	40	94	[58]
8	[MerDABCO‐SO_3_H] Cl (5 mg), H_2_O, 80 °C	20	79	[63]
9	*β*‐Cyclodextrin (20 mol%), US, 50 °C	25	91	[64]
10	CO‐MOF@Ag_2_O (20 w%), H_2_O, 50 °C	4	92	[64]
11	ZnFe_2_O_4_/GA(0.01 g), H_2_O, 100 °C	15	98	[32]
12	Perlite NPs/Met‐Co(II) (5 mg), H_2_O, 100 °C	35	95	–

a) Reaction conditions: ethyl acetoacetate (1 mmol), hydrazin hydrate (1 mmol), 4‐Nitrobenzaldehyde (1 mmol), barbituric acid (1 mmol) under explained conditions.

## 3 Conclusion

This work demonstrates the successful design of a hierarchically structured Perlite NPs/Met‐Co(II) nanocatalyst, characterized by FTIR, XRD, field‐emission scanning electron microscopy (FESEM), EDX, TGA, and BET, confirming effective functionalization and porous architecture. Significant findings include its high efficiency in catalyzing the green, one‐pot multicomponent synthesis of pyrazolopyranopyrimidines, achieving 8795% yields under mild conditions (water, 100 °C, 5 mg catalyst loading) with excellent atom economy and short reaction times. The synergistic combination of the porous perlite support's, CPTES linker's molecular anchoring capability and metformin‐Co(II) complex's as catalytic active sites, establishes this system as a sustainable platform for heterocycle synthesis, aligning with green chemistry principles through energy‐efficient operation, atom economy, and reduced environmental impact compared to conventional homogeneous catalysts. The prepared catalyst can be used for the synthesis of other (bioactive) heterocycles, optimizing for industrial‐scale production with continuous flow systems, scaling up for industrial processes, and evaluating the pyrazolopyranopyrimidine products through advanced bioassays (e.g., MTT assays for anticancer activity) to explore their pharmacological potential, thereby bridging green chemistry with pharmaceutical development and advancing ecofriendly catalytic platforms.

## 4 Experimental Section

4.1

4.1.1

##### Chemicals, Instrumentation, and Analysis

All chemicals and solvents used in this study were purchased from reputable international suppliers such as Sigma–Aldrich and Merck in the highest available purity and were used without further purification. Reaction progress and product purity were monitored using thin‐layer chromatography (TLC) on silica gel 60 F254 plates. Structural analysis of organic compounds was performed using advanced spectroscopic techniques^1^H NMR (400 MHz) spectra was recorded on Bruker Avance; DRX was recorded in deuterated chloroform (CDCl_3_) using tetramethylsilane (TMS) as internal standard. FTIR spectra were measured using KBr pellet method in the range of 4000–400 cm^−1^ by Magana 550 Nicolet. Melting points were determined in capillary tubes using a Barnstead Electrothermal MK3 apparatus (uncorrected). Various characterization techniques were employed for catalyst analysis: XRD patterns were recorded using a Bruker D8‐Advance instrument with Cu K*α* radiation (*λ *= 1.5406 Å). Morphological evaluation was performed by field emission scanning electron microscopy (FESEM, ZEISS Sigma model) at an accelerating voltage of 15 kV. BET analysis was done using Belsorp Mini II instrument. Thermal stability of samples was investigated using TGA, on a BAHR STA 503 devise.

##### Catalyst Preparation: Synthesis of Perlite Nanoparticles (Perlite NPs)

The perlite nanoparticles were prepared through a sequential calcination acid treatment protocol. Initially, crystalline perlite (5 g) was thermally activated by calcination at 500 °C for 1 h. The calcined material then underwent acid washing using HCl (2 M, 150 ML) under reflux conditions for 24 h to remove impurities and enhance porosity. The resulting product was thoroughly washed with distilled water to neutral pH, followed by a final calcination step at 700 °C for 2 h to achieve complete structural reorganization. This process yielded high‐purity perlite nanoparticles as a fine white powder.^[^ ^2^ ^,^ ^7^ ^,^ ^8^ ^]^

##### Functionalization of Perlite NPs with Chloropropyltriethoxysilane (Perlite NPs/CPTES)

The perlite nanoparticles (6.0 g) were first dispersed in anhydrous toluene (20 mL) using ultrasonic treatment (30 min) to achieve complete deagglomeration sample. Chloropropyltriethoxysilane (CPTES, 1.5 mL) was added dropwise to obtained suspension under nitrogen atmosphere. The reaction was proceeded at 70 °C for 18 h. After the time, the Perlit NPs/CPTES were separated by centrifugation (10,000 rpm, 10 min) with alternating washes with deionized water and ethanol (3 times each) to ensure complete removal of remained silanes. Finally, the obtained product was dried at 60 °C for 12h.

##### Attachment of Metformin on Perlite NPs/CPTEs (Perlite NPs/CPTES/Met)

The Perlite NPs/CPTES nanocomposite (2.0 g) was dispersed in anhydrous toluene (10 mL) via ultrasonic treatment (15 min) to achieve deagglomerate suspension. Then, metformin (0.2 g) and sodium bicarbonate (0.2 g) were sequentially added, followed by additional sonication (15 min). The reaction mixture was then heated at 90 °C under reflux for 24 h under nitrogen atmosphere. After the time and cooling to ambient temperature, the resulting Perlite NPs/CPTES/Met nanocomposite was isolated by centrifugation and washed with toluene and acetone to remove any unreacted species. Final drying at 60 °C for 6 h yielded the perlite NPs/CPTES/Met powder.

##### Immobilization of Co(II) on Perlite NPs/CPTES/Met (Perlite NPs/CPTES/Met‐Co(II))

The Perlite NPs/CPTES/Met‐Co(II) nanocatalyst was prepared through a controlled metal coordination process. An aqueous solution of CoCl_2_ · 6H_2_O (0.1 g in 5 mL DI water) was added to the Perlite/CPTES/Met (0.3 g) under sonication for 15 min. The mixture was then subjected to continuous stirring at 25 °C for 24 h to facilitate complete Co(II) complexation with the nitrogen‐rich metformin ligands anchored on the functionalized perlite surfaces. Finally, the obtained product was washed with DI water and dried at 70 °C for 6 h to yield the final catalyst.

##### General Procedure for the Preparation of Pyrazolopyranopyrimidine Derivatives

In a round‐bottom flask, aromatic aldehyde (1 mmol), ethyl acetoacetate (1 mmol, 130 mg), hydrazine hydrate (1 mmol, 50 mg), and barbituric acid or derivative (1 mmol, barbituric acid: 128 mg; thiobarbituric acid: 144 mg) were added to water (5 mL) along with Perlite NPs/Met‐Co(II) catalyst (5 mg). The mixture was stirred at 100 °C for 30–60 min (monitored by TLC). After completion, the reaction was cooled, the solid product filtered, washed with cold water (2 × 5 mL), and ethanol (5 mL) and recrystallized from ethanol if needed. Products were characterized by ^1^H NMR and ^13^C NMR (DMSO‐*d*_*6*_), FTIR, and melting point.

##### Spectral Data: 4‐(4‐Methoxyphenyl)‐3‐Methyl‐4,8‐Dihydropyrazolo [4^′^, 3^′^:5,6]pyrano[2,3‐d]pyrimidine‐5,7(1H, 6H)‐Dione (5b)

Yellow solid; M.P. 226–228 °C (lit. 228–230 °C);^[^ ^32^ ^]^ ATR‐IRS Ū (cm^−1^) = 3424 (NH), 2927 (C–H sp^2^), 2841 (C–H sp^3^), 1729 (C= O), 1548, 1461 (C = C aromatic), 1436, 1347 (CH_3_), 1172 (C‐O); ^1^H NMR (400 MHz, DMSO‐*d*_*6*_) *δ* (ppm: 13.25 (s, 1H, NH), 10.15 (s, 2H, NH), 6.95 (d, *J *= 8.0 Hz, 2H, ArH), 6.75 (d, *J *= 8.0 Hz, 2H, ArH), 5.37 (s, 1H, CH), 3.7 (s, 3H, CH_3_), 2.26 (s, 3H, CH_3_).

##### 4‐(2‐Chlorophenyl)‐3‐Methyl‐7‐Thioxo‐4,6,7,8‐Tetrahydropyrazolo [4^′^, 3^′^:5,6]pyrano[2,3‐d]pyrimidin‐5(1H)‐One (5h)

Yellow solid; M.P. 222–225 °C (lit. 222–224 °C);^[^ ^63^ ^]^ ATR‐IRS Ū (cm^−1^) = 3414 (NH), 3022 (C–H sp2), 2860 (C–H sp3), 1615 (C=O), 1586, 1471 (C=C aromatic), 1228 (C‐O), 1156 (C=S), 727 (C‐Cl); ^1^H NMR (400 MHz, DMSO‐d_6_) *δ* (ppm): 13.47 (s, 1H, NH), 11.49 (s, 2H, NH), 7.44 (d, *J *= 8.0 Hz, 1H, ArH), 7.32 (d, *J *= 8.0 Hz, 1H, ArH), 7.26‐ 7.17 (m, 2H, ArH). 5.52 (s, 1H, CH), 2.25 (s, 3H, CH_3_).

##### 3,6,8‐Trimethyl‐4‐Phenyl‐4,8‐Dihydropyrazolo [4^′^, 3^′^:5,6]pyrano[2,3‐d]pyrimidine‐5,7(1H, 6H)‐Dione (5l)

Yellow solid; Mp. 191–193 °C (lit. 192–193 °C);^[^ ^40^ ^]^ ATR‐IRS Ū (cm^−1^) = 3453 (NH), 3052 (C–H sp^2^), 2925 (C–H sp^3^), 1683 (C=O), 1573, 1556 (C=C aromatic), 1446, 1385 (CH_3_), 1142 (C‐O); ^1^H NMR (400 MHz, DMSO‐*d*_*6*_) *δ* (ppm) = 13.53 (s, 1H, NH), 7.18 (t, *J *= 8.0 Hz, 2H, ArH), 7.10 (t, *J *= 8.0 Hz, 1H, ArH), 7.04 (d, *J *= 8.0 Hz, 2H, ArH), 5.60 (s, 1H, CH), 3.19 (s, 6H, 2NCH_3_), 2.28 (s, 3H, CH_3_).

## Conflict of Interest

The authors declare no conflict of interest.

## Author Contributions

**Jalal Reihani Shurbakhlu**: investigation: (supporting), **Leila Moradi**: supervision: (lead).

## Data Availability

The data that support the findings of this study are available in the supplementary material of this article.
